# Methods to Enhance the Metabolic Stability of Peptide-Based PET Radiopharmaceuticals

**DOI:** 10.3390/molecules25102314

**Published:** 2020-05-14

**Authors:** Brendan J. Evans, Andrew T. King, Andrew Katsifis, Lidia Matesic, Joanne F. Jamie

**Affiliations:** 1 Department of Molecular Sciences, Macquarie University, Sydney, NSW 2109, Australia; brendan.evans@hdr.mq.edu.au (B.J.E.); andrew.king2@hdr.mq.edu.au (A.T.K.); 2 Department of Molecular Imaging, Royal Prince Alfred Hospital, Camperdown, NSW 2050, Australia; andrewk@nucmed.rpa.cs.nsw.gov.au; 3 Australian Nuclear Science and Technology Organisation (ANSTO), Lucas Heights, NSW 2234, Australia; lidia.matesic@ansto.gov.au

**Keywords:** radiopharmaceuticals, peptides, positron emission tomography, proteolysis, metabolic stability

## Abstract

The high affinity and specificity of peptides towards biological targets, in addition to their favorable pharmacological properties, has encouraged the development of many peptide-based pharmaceuticals, including peptide-based positron emission tomography (PET) radiopharmaceuticals. However, the poor in vivo stability of unmodified peptides against proteolysis is a major challenge that must be overcome, as it can result in an impractically short in vivo biological half-life and a subsequently poor bioavailability when used in imaging and therapeutic applications. Consequently, many biologically and pharmacologically interesting peptide-based drugs may never see application. A potential way to overcome this is using peptide analogues designed to mimic the pharmacophore of a native peptide while also containing unnatural modifications that act to maintain or improve the pharmacological properties. This review explores strategies that have been developed to increase the metabolic stability of peptide-based pharmaceuticals. It includes modifications of the *C*- and/or *N*-termini, introduction of d- or other unnatural amino acids, backbone modification, PEGylation and alkyl chain incorporation, cyclization and peptide bond substitution, and where those strategies have been, or could be, applied to PET peptide-based radiopharmaceuticals.

## 1. Introduction

Positron emission tomography (PET) is a nuclear medicine imaging technique for the non-invasive quantitative measurement of specific biochemical, physiological, and pharmacological processes in vivo [1]. PET is useful in the diagnosis and staging of neurological, cardiovascular, and various oncology-based diseases [2]. PET imaging is achieved by administering a patient with a PET radiopharmaceutical which will localize into organs and/or tissues that express the desired biological target. Once localized, the distribution of the PET radiopharmaceutical throughout the body can be imaged with a PET scanner and a diagnosis can be made. PET radiopharmaceuticals are biologically active molecules that are labeled with positron-emitting radionuclides such as fluorine-18, gallium-68, or copper-64. A key component of a PET radiopharmaceutical is the targeting entity, which is designed to possess a pharmacophore that has high affinity and specificity towards a desired biological target present in an organ and/or tissue that is associated with a specific disease or malignancy [3]. The targeting entities of radiopharmaceuticals were initially developed as biologically active small molecules, as is the case for the most widely used PET radiopharmaceutical [^18^F]fluorodeoxyglucose [4]. However, in recent years there has been a rapid development in the use of targeting entities developed from biologics, such as peptides, proteins, antibodies, and antibody fragments for the use as PET radiopharmaceuticals [5,6,7].

### Peptides as Radiopharmaceuticals

**Figure 1:**
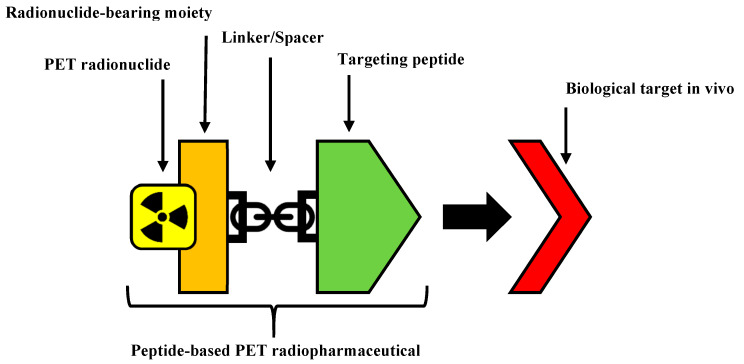
The structural components of a peptide-based positron emission tomography (PET) radiopharmaceuticals.

The structure of a peptide-based radiopharmaceutical (Figure 1) typically contains the following components: a peptide to act as the targeting entity, a linker, a radionuclide bearing moiety, and a PET radionuclide. The linker is sometimes an optional component of a radiopharmaceutical that is incorporated to facilitate the conjugation of the targeting peptide and the radionuclide bearing moiety, and/or improve its pharmacokinetics such as by increasing metabolic stability or manipulating biodistribution [8,9,10,11]. The linker can also be used as a spacer to distance bulky portions of a radiopharmaceutical, such as chelators from the bioactive portions, to reduce steric interference and maintain high binding affinity [12].

There are many advantages offered by utilizing peptides as the targeting entity in radiopharmaceuticals, especially in the field of oncology. These peptide-based radiopharmaceuticals can take the form of radiopharmaceuticals for the diagnosis of diseases by imaging the biological target associated with the disease in specific tissues, or as radiotherapeutics for the treatment of cancers by subjecting the tissue to localized ionizing radiation. Furthermore, the opportunity to exploit the same targeting agent with either an imaging or therapeutic radionuclide has given rise to the field of ‘Theranostics’ in nuclear medicine [13].

A key advantage of peptide-receptor targeting peptide-based radiopharmaceuticals is the higher density of peptide receptors expressed on tumor cells than in normal tissues, thus specific receptor-binding radiolabeled peptides can be designed to enable the efficient visualization and treatment of various tumors [14]. Due to the relatively small size of peptides compared to other biologics, such as proteins and antibodies, peptides often exhibit rapid pharmacokinetics, with the ability to efficiently penetrate tumors, fast clearance from the bloodstream and non-target tissues, and are not immunogenic [5,14,15,16]. Peptides can also be readily synthesized using conventional peptide synthesizers, and any desired modifications to the structure can be easily engineered by making the appropriate changes to the peptide sequence during synthesis and/or by adding other structural modifications after synthesis [9,17,18].

**Figure 2:**
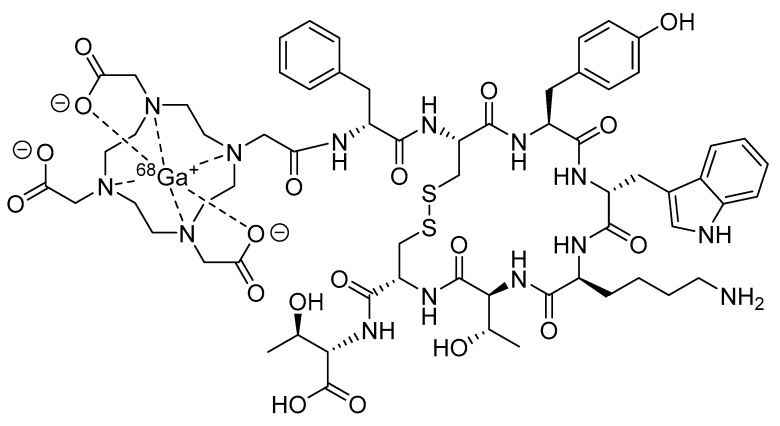
Structure of [^68^Ga]DOTATATE (NETSPOT^®^).

Consequently, the last 20 years have seen an explosive growth in the development of radiolabeled peptides for targeted diagnostic imaging and therapy. While radiolabeled peptides have been applied to various molecular imaging modalities that use nuclear probes, such as scintigraphy and single-photon emission computed tomography (SPECT), the superior image quality and quantitative data available from PET have resulted in a significant amount of research being devoted to the development of PET radiolabeled peptides [19]. Recently, [^68^Ga]DOTATATE, also known as NETSPOT^®^ (Figure 2), was the first radiolabeled peptide for PET imaging to receive regulatory approval from the Food and Drug Administration (FDA) [20].

Despite the advantages offered by peptides, there are several challenges inherent to the use of peptides in drug design and development, including for imaging and therapy. The most significant of these is that unmodified peptides often possess prohibitively short half-lives in vivo, primarily due to rapid proteolytic degradation in the blood, liver, and kidneys by endogenous proteases [21]. This liability results in a short duration of in vivo activity, poor bioavailability, and has significantly limited their application in drug development [22].

## 2. Challenges Faced by Peptide-Based Radiopharmaceuticals In Vivo

The rate at which a drug is metabolized and removed from the body determines its biological half-life [23]. In the case of radioactive drugs, such as PET radiopharmaceuticals, the physical half-life is determined by the incorporated radionuclide [23]. Thus, to achieve optimal results, the localization process of a radiopharmaceutical has to be fast relative to its biological and physical half-lives, such that the drug will localize to the target with adequate time for imaging or therapy before degrading below an effective activity and/or concentration [24]. In the case of peptide-based radiopharmaceuticals, the two most significant forms of degradation in vivo that impact the efficacy of the drug are loss of the radionuclide and degradation of the conjugated peptide. The premature degradation of peptide-based radiopharmaceuticals in vivo is of pressing concern since these drugs are typically administered in doses that are significantly smaller than conventional drugs and are therefore especially vulnerable to having their efficacy significantly disrupted by any amount of degradation in vivo.

### 2.1. Loss of the Radionuclide

Many radiopharmaceuticals are labeled with radiometals (e.g., technetium-99m, gallium-68, and copper-64). Ensuring that the radiometal is not lost from the radiopharmaceutical in vivo is of critical concern when developing these drugs as the free radiometal may exhibit high toxicity and cause significant damage to the body [25]. In the case of fluorine-18 labeled radiopharmaceuticals, in vivo radiodefluorination results in the release of free [^18^F]fluoride ions that can readily accumulate into the calcium-rich fluorophilic bones of the body [26]. Radiodefluorination and in vivo metabolism of [^18^F]radiopharmaceuticals also present major challenges to imaging studies as non-specific uptake of free [^18^F]fluoride ions and [^18^F]metabolites can lead to a degradation of the signal to noise ratio [27]. Further information on the radiodemetallation and radiodefluorination of radiopharmaceuticals, and the strategies that have been applied to mitigate these issues, are beyond the scope of this review but have been thoroughly reviewed elsewhere [28,29,30].

### 2.2. Degradation of the Peptide

The major challenge of using peptides in the active component of a pharmaceutical is that naturally occurring peptides are usually rapidly degraded in vivo [21,22]. Compared to other biologics, such as proteins and antibodies, peptides are generally more susceptible to enzymatic and/or chemical degradation. One of the key reasons a peptide sequence can also be susceptible to proteolytic degradation is due to its backbone containing a recognition motif for an endogenous protease [31]. In addition to proteolytic degradation, peptide bonds can also undergo spontaneous degradation under physiological conditions when particularly labile sequence motifs are present [32].

In peptide-based radiopharmaceuticals, degradation of the conjugated peptide will significantly disrupt its ability to localize to the target tissue, and the subsequent radioactive metabolites will undergo non-specific binding to other tissues and/or be rapidly cleared from the body. In the case of radiopharmaceuticals, this will reduce imaging sensitivity due to increasing background radiation [33]. In radiotherapeutics, this can result in insufficient irradiation of the target tissue while increasing the irradiation of non-target tissues [34,35].

Another point to consider is that amide bonds are often used to conjugate the radionuclide bearing moiety to the biomolecule or linker and these bonds are also susceptible to the same degradation pathways as peptide bonds via proteases and amidases [36]. As a result of these challenges, most peptides for use in radiopharmaceuticals are synthetically modified to minimize metabolic degradation in vivo [15,37].

## 3. Increasing the In Vivo Stability of Peptide-Based Radiopharmaceuticals

The peptide amide bond represents the central repeating structural element of peptides and proteins. This bond possesses partial double bond character, which is one of the key attributes that contributes to the rigidity of peptide chains. Its ability to form hydrogen bonds also makes the peptide bond play a crucial role in its recognition by and interactions with other proteins. Normally, the peptide amide bond is stable to hydrolysis, requiring harsh conditions involving concentrated acids or bases at increased temperatures [38,39]. However, the peptide bond can be readily cleaved under mild conditions at or even below room temperature in the presence of an appropriate protease or peptidase [39,40].

**Figure 3:**
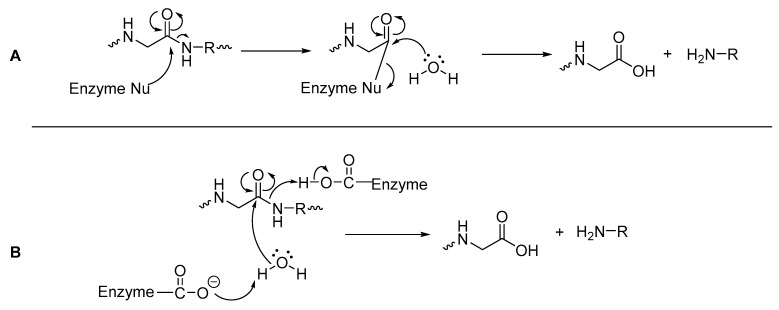
General mechanisms of hydrolysis via a peptidase (**A**) with nucleophilic amino acids; (**B**) with acidic amino acid residues [43,44].

Peptidases can either be classified as exopeptidases, which specifically hydrolyze the *C*- or *N*-termini of a peptide, or as endopeptidases which are capable of hydrolyzing amide bonds within a peptide [41,42]. Mechanistically, hydrolysis of an amide bond with a peptidase occurs via a nucleophilic attack at the carbonyl carbon of the amide bond (Figure 3), with its pathway dependent on the amino acids present in the peptidase’s active site [43]. Peptidases that have nucleophilic amino acids residues such as cysteine and serine in their active site, can attack the carbonyl carbon of the amide/peptide bond and form an acylated enzyme (as a thioester for cysteine or ester for serine), which is more vulnerable to attack by water than the original peptide bond (Figure 3A). Often these peptidases also have histidine and aspartic acid or glutamic acid within the active site, as a ‘catalytic triad’ [43,44]. With peptidases that have amino acid residues such as aspartate/aspartic acid or glutamate/glutamic acid (but no serine or cysteine) in their active site, these residues directly assist a water molecule in its nucleophilic attack of the carbonyl carbon of the amide bond, leading to direct hydrolysis of the amide bond (Figure 3B) [43].

Several strategies have been developed to synthesize peptide analogues in which vulnerable peptide bonds are either modified or obscured such that they are no longer targeted by proteolytic enzymes. The goal of this is to generate metabolically stable peptide analogues that maintain the biological activity of the original peptide. This review is focused on strategies to enhance metabolic stability of PET peptide-based radiopharmaceuticals. It includes modifications of the *C*- and/or *N*-termini, introduction of d- or other unnatural amino acids, backbone modification, PEGylation and alkyl chain incorporation, cyclization, and peptide bond substitution. While some of the examples discussed in this review have only been applied to SPECT radiopharmaceuticals, radiotherapeutics, or non-radioactive peptide-based pharmaceuticals, the strategies are applicable also to PET peptide-based radiopharmaceuticals.

### 3.1. d-Amino Acids

**Figure 4:**
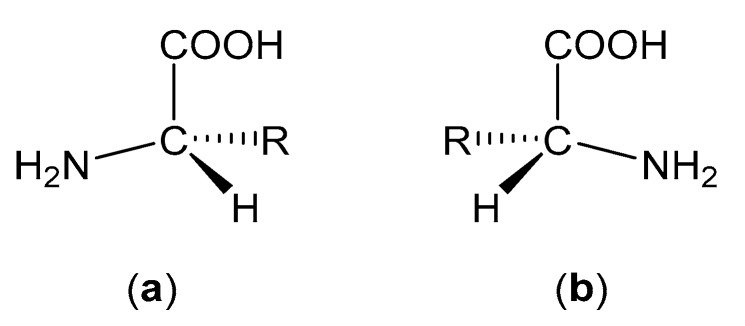
(**a**) Structure of an l-amino acid. (**b**) Structure of a d-amino acid.

Apart from glycine, all amino acids possess chirality and can therefore exist in either levorotatory (l) or dextrorotatory (d) forms (Figure 4). However, nature has proven itself remarkably homochiral and most amino acids are found in their l-form in mammalian systems. d-Amino acids can be found in nature (e.g., in some species of frogs and bacteria), but these cases are exceedingly rare [45].

**Figure 5:**
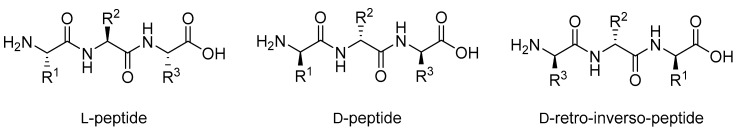
Structure of an l-peptide and its d-peptide analogue and d-retro-inverso-peptide analogue.

The fundamental differences in chirality between l- and d-amino acids means that peptides built from d-amino acids are not recognized by many proteins, including proteases [46]. The result of this lack of recognition is that while most l-peptides are vulnerable to enzymatic degradation in vivo [22], analogous d-peptides are highly resistant to degradation by proteases and have low immunogenicity [47,48]. The simple substitution of all l-amino acids in a peptide with d-amino acids, however, is generally an ineffective strategy as the resulting changes in peptide conformation and side chain orientation can prevent the correct binding geometry and thus destroy target binding [49,50,51,52]. A common solution to this issue is retro-inversion, which constitutes reversing the d-peptide’s sequence (Figure 5). This approach has proven successful in increasing the biological activity of some unstructured d-peptides by restoring the native l-amino acid side chain angles [53,54]. However, in structured peptides, retro-inversion is not enough to overcome the conformational changes caused by the introduction of d-amino acids [46]. For example, left-handed helices in d-peptides remain left-handed even after sequence reversal in retro-inversion, while helices in l-peptides are always right-handed; this difference results in a significant decrease in the binding efficiency of peptides to their biological target [51,55].

Within the Protein Data Bank (PDB), approximately 62% of protein–protein interactions involve helical structures [56]. Furthermore, approximately 80% of peptide drugs approved by the FDA contain helical structures [57]. Due to this, retro-inversion may not be able to be applied to most clinically interesting peptides to correct for the introduction of d-amino acids. However, the benefits offered by d-amino acids can also be conferred to a peptide without substituting every amino acid with its d-amino acid equivalent. For example, substituting the *N*-terminal l-amino acid of most proteins with the corresponding d-amino acid can significantly increase in vivo stability by preventing recognition of the *N*-terminus of the protein by proteases [58].

**Figure 6:**
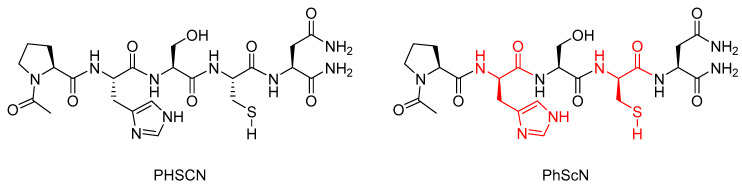
Structures of PHSCN and PhScN peptides, with the d-amino acids highlighted in red [59].

Research conducted by Donna et al. showed that the l-histidine and l-cysteine residues in the α5β1 integrin inhibitor peptide Ac-PHSCN-NH_2_ (PHSCN) could be replaced with their d-stereoisomers to give the mixed chirality peptide Ac-PhScN-NH_2_ (PhScN) (Figure 6) [59]. They found that the mixed chirality peptide PhScN showed significantly improved potency as an inhibitor of α5β1 integrin-mediated invasion of naturally occurring basement membranes in vitro, with IC_50_ values of 0.097 pg/mL and 0.113 pg/mL for DU 145 and PC-3 cells, respectively, compared to 2600 pg/mL and 16,627 pg/mL for the unmodified analogue PHSCN [59]. Donna et al. proposed that the inclusion of the d-amino acids, d-histidine, and d-cysteine in the PhScN peptide could greatly increase systemic stability of the peptides compared to the unmodified analogue due to the resistance these unnatural amino acids show against endoproteinases [59].

**Figure 7:**
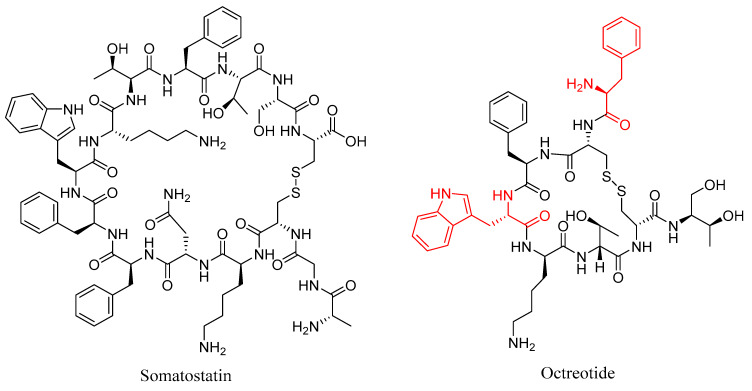
Structures of somatostatin and octreotide, with the d-amino acid modifications on octreotide highlighted in red.

One of the best-known examples of targeted peptide modification for increasing the metabolic stability of a peptide is octreotide (Figure 7); a peptide-based drug developed from the endogenous hormone somatostatin [60]. Somatostatin (Figure 7) is a 14 amino acid long cyclic peptide that inhibits the secretion of a growth hormone. Somatostatin receptors are also found in high concentrations in various neuroendocrine tumors [60,61]. The clinical application of somatostatin is severely limited by its in vivo half-life of only 1–2 min in human plasma due to rapid enzymatic degradation [60]. This limitation spurred the development of somatostatin analogues with more useful properties, including octreotide, in which the somatostatin amino acid sequence was shortened from 14 to 8 amino acids, the l-tryptophan residue was replaced with d-tryptophan, and the *N*-terminal l-amino acid was replaced with a d-amino acid [60]. These modifications increased the in vivo half-life to 1.5 h in human plasma, while maintaining a high binding affinity for the somatostatin receptor subtype SSTR2, with an IC_50_ value of 0.56 nM for octreotide compared to 0.23 nM for the native somatostatin [60,62]. The improved half-life and maintained potency offered by octreotide and derivatives thereof led to their radiolabeling with a variety of radioisotopes for use in the diagnosis and treatment of various neuroendocrine tumors, including indium-111 for SPECT imaging [61,63], carbon-11 [64] and gallium-68 [65] for PET imaging, and yttrium-90 [61] and lutetium-177 [66] for radiotherapy.

**Figure 8:**
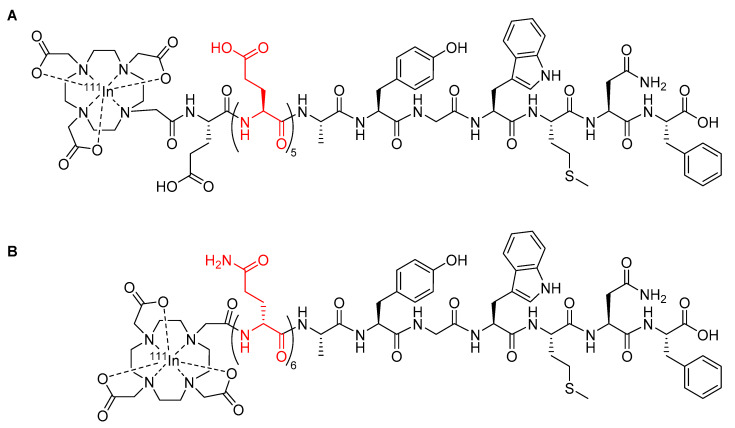
(**A**) Minigastrin analogue [^111^In-DOTA]MG0 with the five l-glutamic acids linker highlighted in red [67]; (**B**) minigastrin peptide radiopharmaceutical developed by Kolenic-Petial et al., with the six d-amino acids linker highlighted in red [68].

Radiopharmaceuticals based upon the minigastrin peptide have been developed for the purposes of imaging and therapy to target cholecystokinin 2 receptors overexpressed in a variety of thyroid, lung, and ovarian tumors [67]. However, the clinical utility of early minigastrin radiopharmaceuticals was compromised due to high kidney retention, which can cause nephrotoxicity [67,68]. For example, the minigastrin radiopharmaceutical [^111^In-DOTA]MG0 (Figure 8A) showed a kidney retention of approximately 127% ID/g [67]. To address the issue of kidney retention, new minigastrin analogues were developed with a decreased number of glutamic acid residues to reduce the negative charge on the peptide [68]. A minigastrin analogue with the five glutamic acid residues in the linker deleted from the sequence of [^111^In-DOTA]MG0 had a significantly reduced kidney retention of approximately 0.3% ID/g [69], but it also had a decreased metabolic stability in human serum, with a mean half-life of only 2 h compared to 72.6 h for [^111^In-DOTA]MG0, which ultimately reduced its suitability for clinical use [69]. Kolenic-Petial et al. found that the half-life of these minigastrin analogues could be improved by inserting a linker of non-ionic d-amino acids into the structure [68]. The authors further demonstrated the metabolic stability of the minigastrin analogue could be improved by increasing the length of the linker, with a linker comprised of six d-glutamine residues proving to be optimal and resulting in a half-life in human serum of approximately 495 h (Figure 8B) [68].

While the research of Kolenic-Petail et al. [68] discussed above utilized indium-111 for SPECT imaging, the benefits achieved from utilizing d-amino acids could be easily carried over to PET studies, for example, by chelating a PET radiometal such as gallium-68 in place of indium-111 or by utilizing d-amino acids in the linker between a peptide and a radiofluorinated prosthetic group.

### 3.2. β-Amino Acids

β-Amino acids are widely found as biologically active natural products produced by plants [70], microorganisms [71], and marine organisms [72], but relatively few β-amino acids are found in mammalian systems [73]. The incorporation of single or multiple β-amino acids into peptides is of increasing interest in the pharmaceutical field as they can enhance in vivo metabolic stability [74,75,76,77,78] and potency [74,79]. This has been attributed to their different electronic environments and backbone/side chain configurations, compared to their α-amino acid analogues, decreasing recognition by proteases [80].

**Figure 9:**
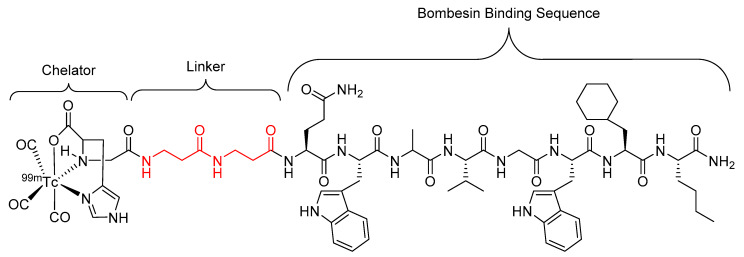
Bombesin-based peptide radiopharmaceutical investigated by Garayoa et al., with the βAla–βAla linker highlighted in red [81].

Garayoa et al. showed that the incorporation of a β-alanine-β-alanine (βAla-βAla) linker into a human tumor targeting bombesin peptide radiopharmaceutical (Figure 9) resulted in a two-fold increase in metabolic stability against proteolytic degradation and no decrease in receptor affinity when compared to the unmodified structure (sans linker) in studies performed using in vitro tumor cell cultures [81]. However, when the studies were performed in human plasma, the analogue with the βAla–βAla linker showed decreased metabolic stability with a half-life of 10 h compared to 16 h for the unmodified analogue [81].

**Figure 10:**
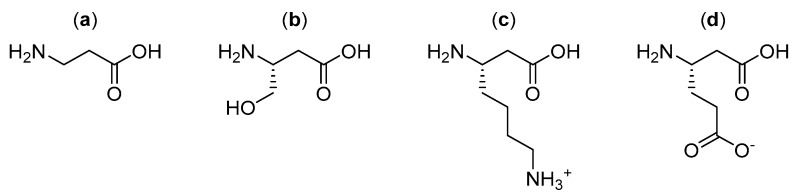
Alternate β-amino acids (**a**) β-alanine; (**b**) β^3^-homoserine; (**c**) β^3^-homolysine; and (**d**) β^3^-homoglutamic acid investigated by Garayoa et al. for use as linkers [17].

Further research by Garayoa et al. investigated alternative β-amino acid linkers (Figure 10) for the same bombesin peptide radiopharmaceutical as shown above (Figure 9), with a focus on β-amino acids that could hold a charge once incorporated into the final structure of the radiopharmaceutical [17]. The linkers were constructed from a combination of two or three of the selected β-amino acids in sequence. It was found that the bombesin analogue modified with β^3^-homoglutamic acid (Figure 10d) in the linker, with one single negative charge, showed a significant increase in tumor uptake and tumor-to-tissue ratio, but did not increase metabolic stability compared to the previously developed radiopharmaceutical (Figure 9) [17].

**Figure 11:**
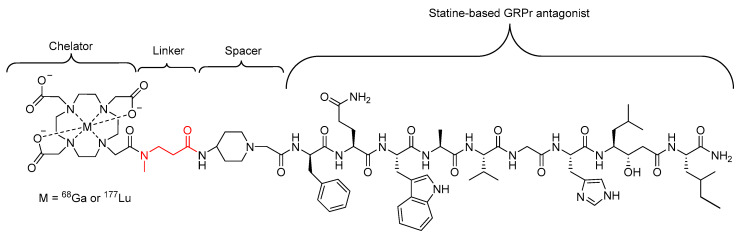
Statine-based GRPr antagonist radiopharmaceutical investigated by Popp et al., with the *N*-methylated β-alanine linker highlighted in red [82].

In a similar study carried out by Popp et al., a single β-alanine amino acid, with a methylated nitrogen, was used as a linker in a statine-based GRPr-antagonist radiopharmaceutical (Figure 11) designed to target receptors on the surface of several human tumors [82]. The introduction of the *N*-methylated β-alanine did not disrupt the binding affinity and presented a similar in vivo stability in mice compared to the unmodified compound, with approximately 50% of the activity in the bloodstream representing intact compound 15 min post injection in the case of both compounds [82].

The research undertaken by Garayoa et al. [17,81] and Popp et al. [82] aimed to demonstrate that unnatural β-amino acids could be introduced into the structure of peptide-based radiopharmaceuticals to increase metabolic stability. While neither author achieved a significant increase in metabolic stability, they did produce compounds that maintained metabolic stability and binding affinity close to the unmodified analogue. This indicates that there is room for improvement for future peptide-based radiopharmaceuticals that incorporate unnatural β-amino acids.

**Figure 12:**
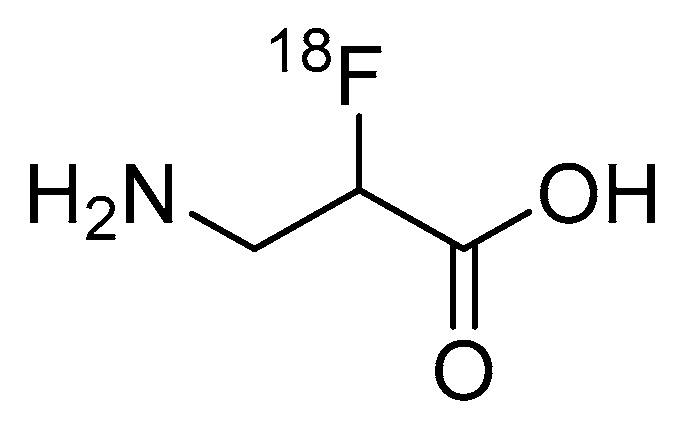
Fluorine-18 labeled β-alanine synthesized by Schjoeth-Eskensen et al. [83].

While the research of Garayoa et al. only used the SPECT radionuclide technetium-99m, it should be apparent that the introduction of β-amino acids can easily be applied to PET studies through, for example, chelating PET radiometals or incorporating β-amino acids into peptide-based PET radiopharmaceuticals. Despite the potential benefits to pharmacological properties, there are currently no examples in the literature of β-amino acids being applied to improve the metabolic stability of peptide-based fluorine-18 labeled radiopharmaceuticals. Interestingly, the research by Schjoeth-Eskensen et al. successfully demonstrated the radiolabeling of the α-carbon of β-alanine with fluorine-18 (Figure 12), but no stability studies or subsequent conjugation to a peptide were performed [83].

### 3.3. N-Methylation

**Figure 13:**
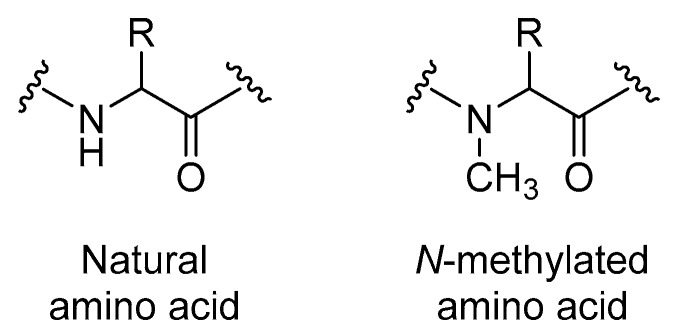
Structures of natural compared to *N*-methylated amino acids.

Peptides can be modified through *N*-methylation (also known as *N*-alkylation). This method constitutes substituting one or more NH groups in a peptide backbone with *N*-methyl substituents (Figure 13). *N*-Methylation can confer several benefits compared to their unmodified analogues, including enhanced protease resistance, membrane permeability, and biological activity [84].

**Figure 14:**
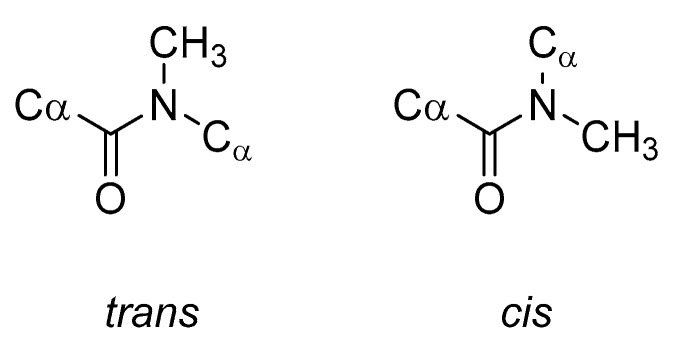
Comparison of the *trans* and *cis* conformations of *N*-methylated peptide bonds.

*N*-Methylation of a peptide backbone replaces a hydrogen bond donor (NH) with a potential steric clash (NCH_3_), thus eliminating some inter- and intramolecular hydrogen bonds [85,86,87]. *N*-Methylation can also greatly alter the conformation of the entire peptide. This occurs because the *N*-methyl group will influence the conformational flexibility of both the peptide backbone and the side chains of the residues close to the *N*-methyl amino acids. Of particular note, the energy barrier between the *trans* and *cis* peptide bond conformations (Figure 14) is greatly reduced and consequently the *cis* peptide bond conformation becomes readily accessible [88].

With the overall conformational change that can occur with *N*-methylated peptides, and the change in H-bonding capacity and steric features, *N*-methylation often leads to a decreased affinity of the peptide for the active site of proteases and therefore increased metabolic stability. It has also been found that *N*-methylation of an amide bond adjacent to the cleavage site can confer a greater resistance against enzymatic degradation than *N*-methylation of the amide bond at the cleavage site itself [89]. This behavior may result from *N*-methylation making the *cis* conformation readily accessible and then becoming the preferred conformation of the peptide in vivo. For example, the *cis* conformation may result in portions of the peptide being positioned such that they are now less accessible to proteolytic activity or simply no longer fit into the enzyme binding site, thus increasing the metabolic stability [88]. However, these structural changes may also disrupt intra- and intermolecular hydrogen bonds that may be important for the stabilization of biologically active conformations and for target receptor recognition [90]. Therefore, the use of *N*-methylation for increasing metabolic stability must be balanced against maintaining biological activity against the desired target receptor [91,92].

The endothelin peptides are potent vasoconstrictors. It has been found that highly potent antagonists of endothelin receptors can be developed from the *C*-terminal hexapeptide of endothelin [88]. However, these compounds are generally unstable towards enzymatic proteolysis and consequently have relatively short half-lives, which reduces their utility for clinical use [88]. Wayne et al. found that the *N*-methylation of a single isoleucine residue in the sequence of these previously developed endothelin receptor antagonists could significantly improve metabolic stability [88]. For example, *N*-methylation of the amide bond between the Ile^19^ and Ile^20^ residues increased the half-life in rat intestinal perfusate from 10.6 min to 538 min [88]. This modification also enhanced receptor binding affinity from an IC_50_ of 40 nM in the case of the unmodified compound down to 10 nM [88].

*N*-Methylation has also been effective when combined with other peptide modifications to further modulate the properties of a peptide-based radiopharmaceuticals. For example, as previously discussed in Section 3.2, Popp et al. successfully incorporated an *N*-methylated β-alanine residue as a linker in a statine-based GRPr antagonist radiopharmaceuticals [82].

### 3.4. PEGylation and Alkyl Linkers

**Figure 15:**
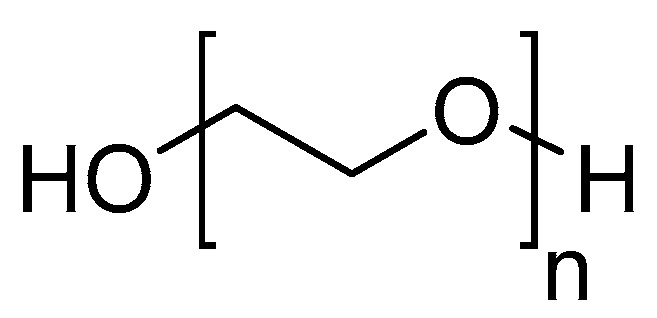
Polyethylene glycol (PEG) structure.

PEGylation defines the process of linking one or more polyethylene glycol (PEG) polymer chains (Figure 15) to a peptide, protein, or non-peptide molecule. PEG possesses useful properties, including high solubility in water and many organic solvents, non-toxicity, and non-immunogenicity, and has been approved by the FDA for human use [93]. Many PEGylated peptide-based radiopharmaceuticals have been developed and shown to possess improved pharmacokinetic properties compared to their unmodified analogues, including increased receptor binding affinity, increased tumor uptake, and decreased kidney uptake [94,95,96,97,98,99,100,101,102]. Other potential improvements that have been achieved though PEGylation include longer circulatory times, increased aqueous solubility [103], and increased metabolic stability by creating steric hinderance that shields the molecule from proteases [104]. PEGylation also increases the molecular mass and size of peptides, which further increases body-residence time due to decreased kidney excretion [105]. The length and shape of PEGs (linear, branched, or dendritic) have been shown to influence the pharmacological properties of the PEGylated peptides and proteins, with branched PEG structures often most effective. This has been postulated to be due to both a greater degree of steric hinderance against proteases and possessing additional sites for conjugation with the target peptide or protein [106].

**Figure 16:**
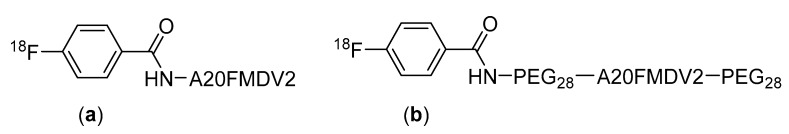
Structures of (**a**) [^18^F]FBA-A20FMDV2 and (**b**) the bi-terminally PEGylated [^18^F]FBA-PEG_28_-A20FMDV2-PEG_28_ [107].

The advantages offered by PEGylation has seen it applied to a variety of peptide-based radiopharmaceuticals. For example, Hausner et al. found that the PEGylation of a radiopharmaceuticals derived from the α_v_β_6_ integrin targeting peptide A20FMDV2, at both the *C*- and *N*-termini, greatly improves its pharmacokinetic properties [107]. When evaluated in mice, the PEGylated peptide showed good metabolic stability with approximately 80% of the compound remaining intact after incubation in mouse serum for 1 h. The modified compound also showed a higher uptake in α_v_β_6_-expressing tumors compared to the unmodified peptide, with a tumor uptake in BxPC-3 cells of 4.7% ID/g compared to 0.69% ID/g at 1 h for the unmodified peptide (Figure 16) [107].

**Figure 17:**
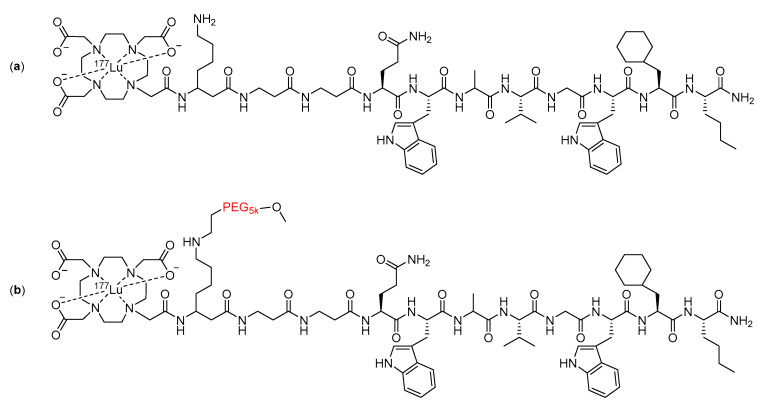
Structure of (**a**) unmodified and (**b**) modified bombesin peptide radiotherapeutics investigated by Dapp et al., with the PEG linker modification highlighted in red [109].

The inclusion of large PEG groups has also been echoed by Dapp and co-workers [108,109], who used a 5 KDa PEG group in the linker of a bombesin peptide radiotherapeutic (Figure 17). The metabolic stability of the compound was assessed in vitro by incubating it in human serum for five days [109]. The inclusion of the PEG into the linker resulted in an increase in the amount of intact radiopharmaceuticals remaining after the five-day incubation, from 14% with the unmodified compound to 52% with the modified compound [109].

**Figure 18:**
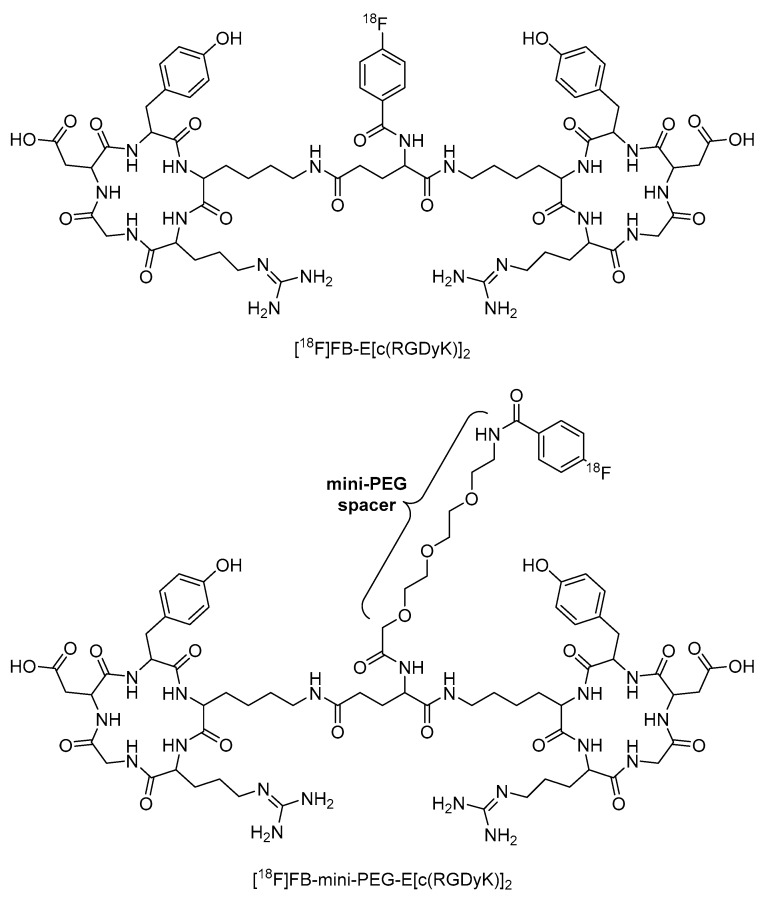
Structures of [^18^F]FB-E[c(RGDyK)]_2_ and [^18^F]FB-mini-PEG-E[c(RGDyK)]_2_ [110].

In another example, Wu et al. incorporated a mini-PEG (three ethylene oxide units) spacer into [^18^F]FB-E[c(RGDyK)]_2_ to produce [^18^F]FB-mini-PEG-E[c(RGDyK)]_2_ (Figure 18). This radiopharmaceutical showed greater radiolabeling yield, reduced renal excretion, and similar tumor uptake compared to the non-PEGylated analogue (Figure 18) [110]. This example also demonstrated that long PEG chains are not always necessary to significantly improve the properties of peptide-based radiopharmaceuticals.

**Figure 19:**
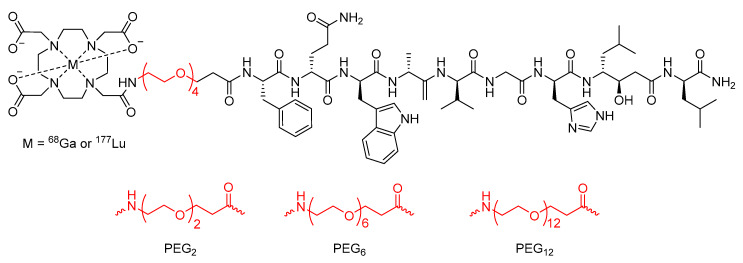
Bombesin-based peptide radiopharmaceutical investigated by the Maecke group, with PEG_4_ chain linker and alternative PEG chain lengths highlighted in red [111,112].

Work conducted by the Maecke group [111] also investigated the effect of PEGylation on the previously developed bombesin analogue DOTA-Bombesin (7–14). Initial studies using a PEG_4_ chain as a linker between the DOTA chelator and the peptide with gallium-68 or lutetium-177 as the radionuclide (Figure 19) found that the modified peptide-based radiopharmaceutical had superior pharmacokinetic properties in the PC-3 tumor-bearing mouse model than previously developed analogues [111]. In a subsequent study, the Maecke group synthesized a series of four lutetium-177 radiolabeled statine-based bombesin antagonist radiopharmaceuticals with PEG chains of different lengths (2, 4, 6, or 12 PEG units) as the linker between the DOTA chelator and the peptide analogue (Figure 19) [112]. The metabolic stability of these compounds was then assessed in human serum, and it was found that the half-life increased as PEG chain length increased up to the PEG_6_ linker (half-life of PEG_2_ = 246 h, PEG_4_ = 407 h, and PEG_6_ = 584 h), but began to decrease with the PEG_12_ linker (half-life of 407 h) [112].

**Figure 20:**
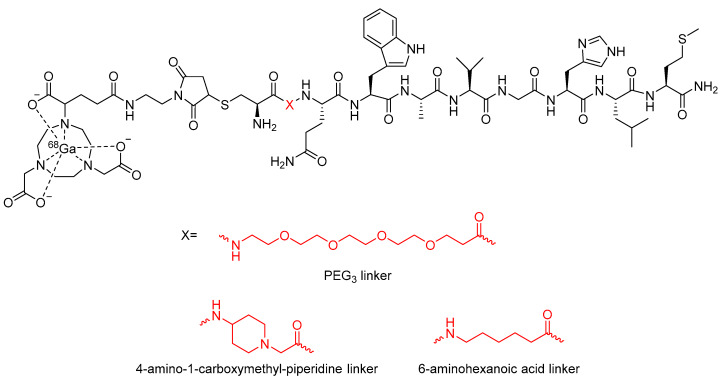
Bombesin-based radiopharmaceuticals investigated by Bacher et al., with the linker location and linkers highlighted in red [113].

**Figure 21:**
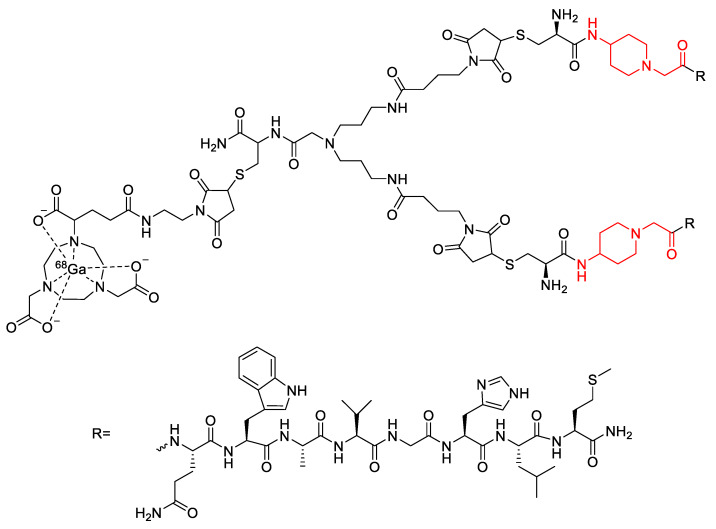
Bombesin-based dimer radiopharmaceutical investigated by Bacher et al., with 4-amino-1-carboxylmethyl-piperidine linkers highlighted in red [113].

In a similar study by Bacher et al., the incorporation of a PEG_3_ linker into a different bombesin-based radiopharmaceutical was explored as a method to increase its metabolic stability (Figure 20) [113]. In vitro stability assays conducted in human serum found that incorporation of the PEG_3_ linker led to a 9% increase in stability compared to the compound not containing a linker [113]. In the same study, Bacher et al. also explored the potential stabilizing effects of other alkyl chains as linkers, including 4-amino-1-carboxymethyl-piperidine and 6-aminohexanoic acid (Figure 20) [113]. However, these analogues were found to decrease metabolic stability by 20% and 7%, respectively, when compared to the radiopharmaceutical without any linker. In contrast to this, it was found that when the 4-amino-1-carboxylmethyl-piperdine linker was incorporated into a dimer of the bombesin-based radiopharmaceutical (Figure 21), there was a 52% increase in stability compared to the dimer without any linker [113]. The incorporation of the 6-aminohexanoic acid linker into the dimer resulted in decreased metabolic stability of the radiopharmaceutical [113].

While beyond the scope of this review, it is interesting to note that PEGylation has also been successfully applied to antibody fragments for the use as PET radiopharmaceuticals, with promising results [114,115].

### 3.5. Peptide Cyclization

**Figure 22:**
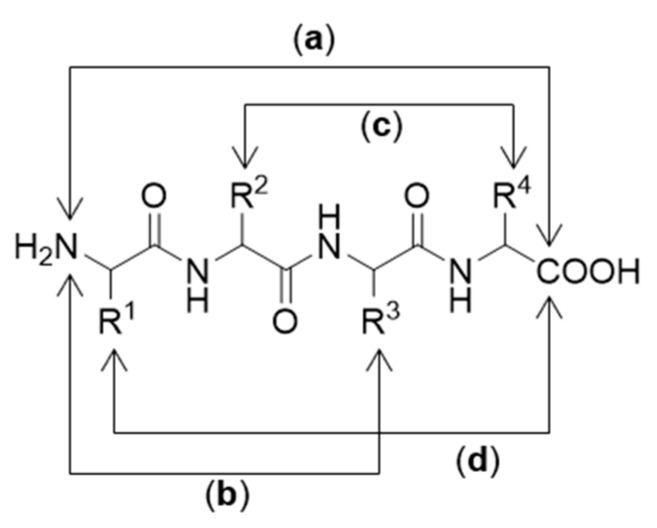
Different cyclization arrangements: (**a**) head-to-tail chain; (**b**) head-to-side chain; (**c**) side chain-to-side chain; (**d**) tail-to-side chain.

Cyclization of a peptide sequence has been found to enhance stability against proteolytic degradation [116]. Cyclization is usually achieved by linking the *C*-terminus to the *N*-terminus of the peptide backbone [117]. However, it can also be advantageous to cyclize peptides by linking the *C*- or *N*-terminus to a side chain or linking one side chain to another side chain [118]. Depending on the desired cyclization site, cyclic peptides can be arranged in several ways, including head-to-tail, head-to-side chain, tail-to-side chain, and side chain-to-side chain (Figure 22) [119].

A distinct advantage of backbone cyclization over other cyclization methods is that cyclization is performed between backbone atoms, leaving the side chains that are usually essential for biological activity untouched [84].

Cyclization of a peptide through the formation of an amide bond between the *C*- and *N*-terminus can prevent degradation of the peptide by exopeptidases [60]. Cyclization of a linear peptide can also be used to increase the peptides’ structural rigidity. This can increase metabolic stability by locking the peptide into a conformation that is less susceptible towards proteolytic enzymes (conformational constraints and/or selective molecular recognition). It can also be used to increase biological activity by locking the peptide into a more biologically active conformation [118,120,121].

Cyclization has been applied to the RGD (Arg-Gly-Asp) peptide sequence that can be used to target the α_v_β_3_-integrin receptors, which are overexpressed on the surface of various tumor cells [33]. In cyclic RGD systems, the RGD peptide sequence is flanked by other amino acids to form a ring system that presents the RGD sequence in a specific conformation. Cyclic RGD systems are more potent, specific, and resistant to proteolysis than their linear analogues [122]. Consequently, cyclic RGD systems have been widely used in the development of various peptide-based PET radiopharmaceuticals [19,123].

### 3.6. Substitution of Amides with Sulfonamides

**Figure 23:**
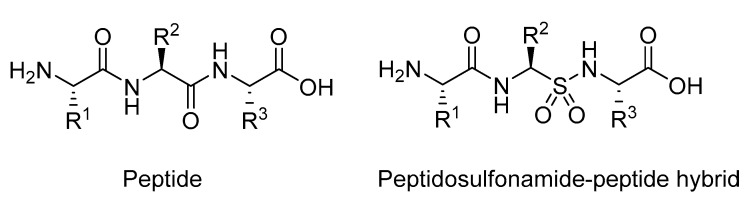
Structure of a regular peptides compared to sulfonamide analogue.

Another strategy that has been applied to increase the metabolic stability of peptide-based drugs is to substitute one or more amide groups in the backbone of a peptide with sulfonamide groups (Figure 23). Incorporation of the sulfonamide moiety into drugs is well known, as exemplified with the classical antibacterial sulfa drugs, and the sulfonamide group is inherently more stable than amides in mammalian systems [124].

**Figure 24:**
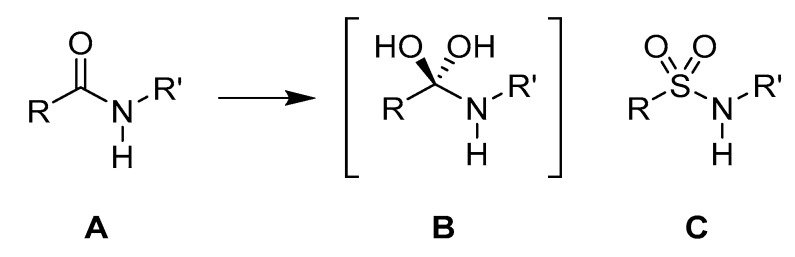
(**A**) Peptide bond structure. (**B**) Transition state for the hydrolysis of the peptide bond. (**C**) Sulfonamide bond as a suggested transition state isostere.

Sulfonamide groups contain a tetrahedral achiral sulfur atom directly bound to two electronegative oxygen atoms. These features with respect to geometry and electronic environment strongly resemble the transition state of peptide bond hydrolysis (Figure 24) [125]. Consequently, peptides containing sulfonamide substitutions have been investigated as transition state isosteres for the use as protease inhibitors [126].

When compared to the amide moiety, the sulfonamide group is a stronger hydrogen bond donor [127] and the sulfonamide N-H is more acidic than the amide N-H, but a weaker hydrogen bond acceptor [127,128,129]. Furthermore, the hydrogen bond accepting character of the sulfonamide moiety is split between two accepting sites due to the two sulfonamide oxygens. These factors can impact the native hydrogen bonding network of the peptide and disrupt the formation of secondary structures. In contrast to the relatively rigid amide peptide bond, the sulfonamide bond is more freely rotatable and the *cis*–*trans* isomerism is not observed [127,130]. This greater rotational freedom allows for the sulfonamide oxygens to assume a variety of positions, where one oxygen occupies a *cis* or *trans* orientation with respect to the amide N-H, while the other oxygen is in neither a *cis* nor *trans* position. This can impede the formation of secondary structures by preventing the proper alignment of hydrogen bonds [127]. These potential disruptions to secondary structure formation have been found to have a greater effect on α-helices and a lesser effect on β-sheets [127].

The replacement of one or more amide bonds along a peptide backbone with sulfonamides has been successfully applied to develop peptidosulfonamide peptide analogues that display increased stability towards proteases compared to their unmodified analogues while also maintaining satisfactory biological activity [127,128,131]. The most common method of applying this strategy is to identify the preferred protease cleavage sites on a peptide and substitute the amides at those locations with sulfonamides. However, it has also been found that the substitution of amides close to cleavage sites can also increase metabolic stability [131]. This may be due to an effect similar to that seen in *N*-methylation where the substitution of the native amide bond with a more flexible bond, in this case a sulfonamide, allows the peptide to take a conformation that prevents proteases accessing the cleavage site [88,90].

**Figure 25:**
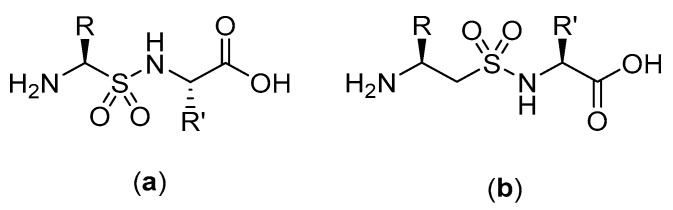
(**a**) Structure of α-peptidosulfonamide-α-peptide hybrid. (**b**) Structure of β-aminosulfonamide-α-peptide hybrid.

The synthesis of a peptide in which all amides in the sequence are substituted with sulfonamides would lead to a peptidosulfonamide oligomer. However, this approach is not wise as α-amino sulfonamides are prone to fragmentation, releasing SO_2_ [132]. This has been addressed by using β-aminosulfonamides, which are more stable than their α-amino analogues (Figure 25) [127].

**Figure 26:**
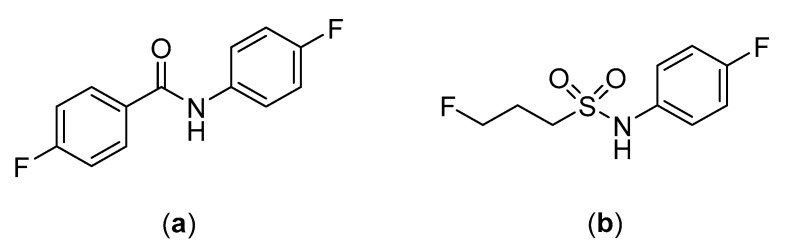
Structures of (**a**) *N*-(4-fluorophenyl)-fluoroacetanilide and (**b**) *N*-(4-fluorophenyl)-3-fluoropropane-1-sulfonamide [36].

The substitution of the amide moiety with sulfonamides is starting to be explored in the development of peptide-based radiopharmaceuticals, including for linking of the peptide to the targeting moiety. For example, common amine-reactive prosthetic groups such as *N*-succinimidyl 4-[^18^F]fluorobenzoate ([^18^F]SFB) and 4-[^18^F]fluorobenzoic acid ([^18^F]FBA) are used to label peptides through the formation of amide bonds with primary amine residues (e.g., *N*-terminus or lysine) present in the peptide backbone [133,134]. While this method of labeling peptides has proven to be convenient, the susceptibility of the resulting amide bonds to hydrolysis in vivo is a potential vulnerability [36,135]. Löser et al. sought to explore this by comparing the metabolic stability of the fluorinated amide, *N*-(4-fluorophenyl)-fluoroacetanilide, and the fluorinated sulfonamide, *N*-(4-fluorophenyl)-3-fluoropropane-1-sulfonamide (Figure 26) [36]. The metabolic stability of both compounds were tested, and after 120 min of incubation in pig liver esterase (the porcine homologue of carboxylesterase), 95% of the *N*-(4-fluorophenyl)-3-fluoropropane-1-sulfonamide compared to only 20% of *N*-(4-fluorophenyl)-fluoroacetanilide remained intact [36]. While the compounds in this study were not complete structural analogues of each other, this research provides evidence of the potential benefits of substituting amide for sulfonamide bonds in radiopharmaceuticals.

## 4. Conclusions

The success of peptide-based PET radiopharmaceuticals, such as NETSPOT^®^, has sparked renewed interest in the development of new PET radiolabeled peptides for targeting diseases in the body. The applicability of new peptide-based radiopharmaceuticals will be influenced to a large extent by their in vivo stability as the inherently poor in vivo stability of natural peptides is one of the biggest challenges in the development of peptide-based radiopharmaceuticals, especially as degradation of the peptide can lead to non-specific binding. There have been several strategies developed to avoid this by modifying natural peptides to enhance their metabolic stability and sometimes other pharmacological properties such as receptor affinity. Effective strategies have included modification of the *C*- and/or *N*-termini, introduction of d- or other unnatural amino acids, backbone modification, PEGylation and alkyl chain incorporation, cyclization and peptide bond substitution. It has also been found that by applying more than one of these modifications in tandem on the same peptide, the different modifications can often work in concert to further enhance metabolic stability. However, no one approach fits all peptides and the decision of which strategy to apply must be made on a case-by-case basis. Consequently, it is rare to find individual studies where several different strategies have been applied to the same peptide to compare their efficacies. While some of the examples discussed in this review have only been applied to SPECT radiopharmaceuticals, radiotherapeutics, or non-radioactive peptide-based pharmaceuticals, the strategies could still be applied to PET radiopharmaceuticals. This could be achieved through simple substitution of a SPECT radiometal with a PET radiometal or by using the modification in the linker between a peptide and the radionuclide bearing moiety in a PET radiopharmaceutical. With the use of the discussed strategies to ensure in vivo stability, the number of successful peptide-based PET radiopharmaceuticals will continue to grow, and their clinical use will continue to expand.
